# 4-Cyano­pyridinium bromide

**DOI:** 10.1107/S160053681202209X

**Published:** 2012-05-19

**Authors:** Wen-Ni Zheng

**Affiliations:** a College of Chemistry and Chemical Engineering, Southeast University, Nanjing 210096, People’s Republic of China

## Abstract

In the title compound, C_6_H_5_N_2_^+^·Br^−^, the pyridine N atom is protonated and involved in an inter­molecular N—H⋯Br hydrogen bond which, together with weak C—H⋯N hydrogen bonds, results in the formation of a chain along the *c* axis. Weak inter­molecular C—H⋯Br inter­actions between pyridine H atoms and Br^−^ anions connect these chains into a network parallel to the *bc* plane.

## Related literature

For the structures and properties of related compounds see: Fu *et al.* (2011*a* ▶,*b* ▶); Dai & Chen (2011 ▶).
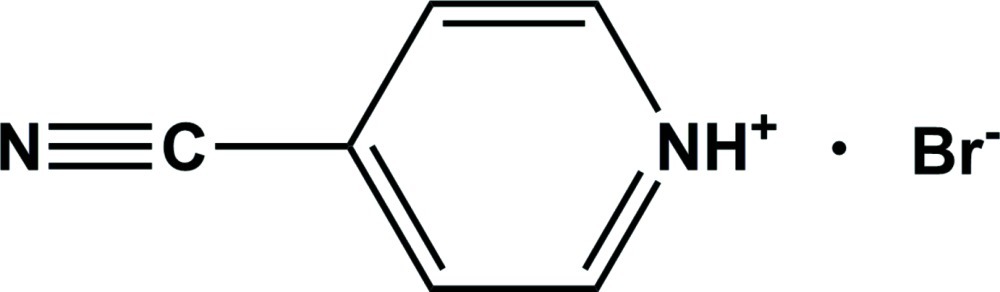


## Experimental

### 

#### Crystal data

C_6_H_5_N_2_^+^·Br^−^
*M* *_r_* = 185.03
Monoclinic, 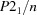

*a* = 7.3918 (5) Å
*b* = 12.2587 (4) Å
*c* = 8.1671 (3) Å
β = 111.720 (1)°
*V* = 687.51 (6) Å^3^
*Z* = 4
Mo *K*α radiation
μ = 5.88 mm^−1^
*T* = 173 K
0.10 × 0.05 × 0.05 mm

#### Data collection

Rigaku Mercury2 diffractometer
Absorption correction: multi-scan (*CrystalClear*; Rigaku, 2005 ▶) *T*_min_ = 0.910, *T*_max_ = 1.000
4771 measured reflections
1579 independent reflections
1296 reflections with *I* > 2σ(*I*)
*R*_int_ = 0.047

#### Refinement

*R*[*F* ^2^ > 2σ(*F* ^2^)] = 0.032
*wR*(*F* ^2^) = 0.066
*S* = 0.95
1579 reflections
82 parameters
H-atom parameters constrained
Δρ_max_ = 0.57 e Å^−3^
Δρ_min_ = −0.92 e Å^−3^

### 

Data collection: *CrystalClear* (Rigaku, 2005 ▶); cell refinement: *CrystalClear*; data reduction: *CrystalClear*; program(s) used to solve structure: *SHELXS97* (Sheldrick, 2008 ▶); program(s) used to refine structure: *SHELXL97* (Sheldrick, 2008 ▶); molecular graphics: *SHELXTL* (Sheldrick, 2008 ▶); software used to prepare material for publication: *SHELXTL*.

## Supplementary Material

Crystal structure: contains datablock(s) I, global. DOI: 10.1107/S160053681202209X/mw2067sup1.cif

Structure factors: contains datablock(s) I. DOI: 10.1107/S160053681202209X/mw2067Isup2.hkl

Supplementary material file. DOI: 10.1107/S160053681202209X/mw2067Isup3.cml

Additional supplementary materials:  crystallographic information; 3D view; checkCIF report

## Figures and Tables

**Table 1:** Hydrogen-bond geometry (Å, °)

*D*—H⋯*A*	*D*—H	H⋯*A*	*D*⋯*A*	*D*—H⋯*A*
N1—H1⋯Br1^i^	0.90	2.26	3.133 (2)	164
C1—H1*A*⋯Br1	0.93	2.88	3.615 (2)	137
C2—H2*A*⋯Br1^ii^	0.93	2.77	3.645 (2)	156
C5—H5*A*⋯N2^iii^	0.93	2.66	3.435 (4)	142

Symmetry codes: (i) 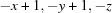; (ii) 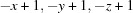; (iii) 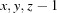.

**Fig. 1.**
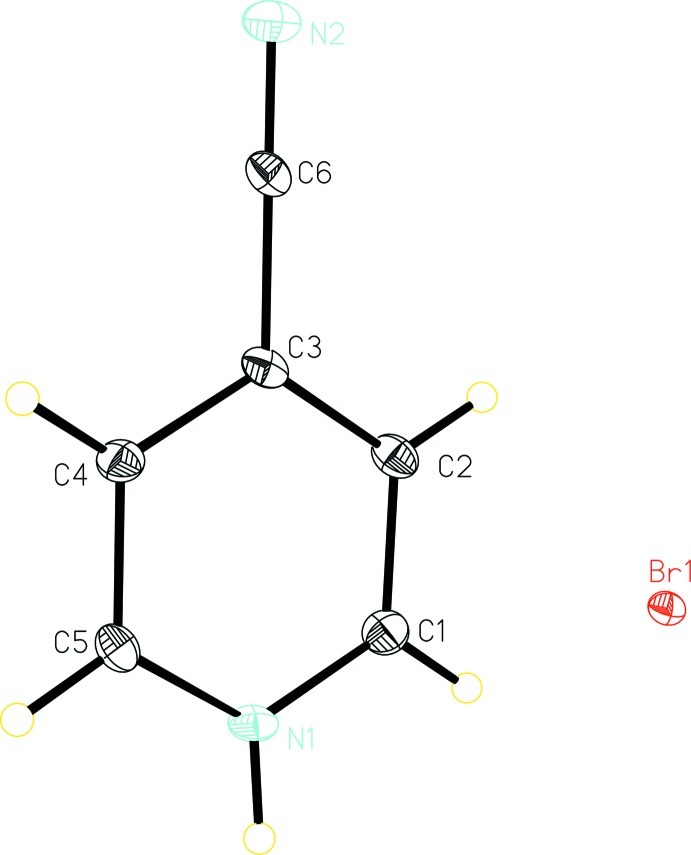
A view of the asymmetric unit with the atomic numbering scheme. The displacement ellipsoids were drawn at the 30% probability level.

**Fig. 2.**
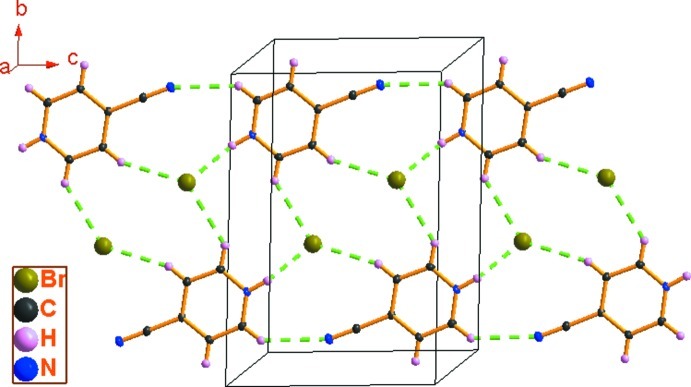
The crystal packing of the title compound viewed along the a axis showing the N—H···Br, C—H···N and C—H···Br interactions (dotted line) in the title compound.

## supplementary crystallographic information

### Comment

Simple organic salts containing strong intermolecular H-bonds have attracted attention as materials which display ferroelectric-paraelectric phase transitions (Fu *et al.*, 2011*a*; Fu *et al.*, 2011*b*). With the purpose of obtaining crystals of organic salts which might undergo such phase transitions, various organic molecules have been studied and a series of new materials have been elaborated (Dai & Chen 2011). Herewith we present the synthesis and crystal structure of the title compound, 4-cyanopyridinium bromide.

In the title compound (Fig. 1), the bond lengths and angles have normal values. The asymmetric unit is composed of one 4-cyanopyridinium cation and one Br^-^ anion. The protonated N atom is involved in a strong N—H···Br hydrogen bond (Table 1) which accompanying the C5—H5A···N2 H-bond generates a linear chain parallel to *c-*axis while weak C1—H1A···Br and C2—H2A···Br1 interactions serve to link the chains into a 3-dimensional layer structure (Fig. 2 and Table 1).

### Experimental

Isonicotinonitrile (20 mmol), aqueous HBr (5 mL, 2 mol/L) and ethanol (50 mL) were added to a 100mL flask. The mixture was stirred at 60° C for 2 h, and then the precipitate was filtrated out. Colourless crystals suitable for X-ray diffraction were obtained by slow evaporation of the filtrate.

### Refinement

All H atoms attached to C atoms were situated into the idealized positions and treated as riding with C–H = 0.93 Å (aromatic) with *U_iso_*(H)=1.2*U_eq_*(C). The positional parameters of the H atom (N) were refined freely. And in the last stage of the refinement, they were restrained with the H—N = 0.90 (2)Å, with *U_iso_*(H)=1.2*U_eq_*(N).

### Crystal data

**Table:**

C_6_H_5_N_2_^+^·Br^−^	*F*(000) = 360
*M**_r_* = 185.03	*D*_x_ = 1.788 Mg m^−^^3^
Monoclinic, *P*2_1_/*n*	Mo *K*α radiation, λ = 0.71073 Å
Hall symbol: -P 2yn	Cell parameters from 1579 reflections
*a* = 7.3918 (5) Å	θ = 3.2–27.5°
*b* = 12.2587 (4) Å	µ = 5.88 mm^−^^1^
*c* = 8.1671 (3) Å	*T* = 173 K
β = 111.720 (1)°	Block, colorless
*V* = 687.51 (6) Å^3^	0.10 × 0.05 × 0.05 mm
*Z* = 4	

### Data collection

**Table:**

Rigaku Mercury2 diffractometer	1579 independent reflections
Radiation source: fine-focus sealed tube	1296 reflections with *I* > 2σ(*I*)
Graphite monochromator	*R*_int_ = 0.047
Detector resolution: 13.6612 pixels mm^-1^	θ_max_ = 27.5°, θ_min_ = 3.2°
ω & φ scans	*h* = −9→9
Absorption correction: multi-scan (*CrystalClear*; Rigaku, 2005)	*k* = −15→15
*T*_min_ = 0.910, *T*_max_ = 1.000	*l* = −10→10
4771 measured reflections	

### Refinement

**Table:**

Refinement on *F*^2^	Primary atom site location: structure-invariant direct methods
Least-squares matrix: full	Secondary atom site location: difference Fourier map
*R*[*F*^2^ > 2σ(*F*^2^)] = 0.032	Hydrogen site location: inferred from neighbouring sites
*wR*(*F*^2^) = 0.066	H-atom parameters constrained
*S* = 0.95	*w* = 1/[σ^2^(*F*_o_^2^) + (0.0255*P*)^2^] where *P* = (*F*_o_^2^ + 2*F*_c_^2^)/3
1579 reflections	(Δ/σ)_max_ = 0.001
82 parameters	Δρ_max_ = 0.57 e Å^−^^3^
0 restraints	Δρ_min_ = −0.92 e Å^−^^3^

### Special details

**Table:**

Geometry. All esds (except the esd in the dihedral angle between two l.s. planes) are estimated using the full covariance matrix. The cell esds are taken into account individually in the estimation of esds in distances, angles and torsion angles; correlations between esds in cell parameters are only used when they are defined by crystal symmetry. An approximate (isotropic) treatment of cell esds is used for estimating esds involving l.s. planes.
Refinement. Refinement of F^2^ against ALL reflections. The weighted R-factor wR and goodness of fit S are based on F^2^, conventional R-factors R are based on F, with F set to zero for negative F^2^. The threshold expression of F^2^ > 2sigma(F^2^) is used only for calculating R-factors(gt) etc. and is not relevant to the choice of reflections for refinement. R-factors based on F^2^ are statistically about twice as large as those based on F, and R- factors based on ALL data will be even larger.

### Fractional atomic coordinates and isotropic or equivalent isotropic displacement parameters (Å^2^)

**Table:**

	*x*	*y*	*z*	*U*_iso_*/*U*_eq_	
Br1	0.61085 (4)	0.41149 (2)	0.32517 (4)	0.01972 (12)	
N1	0.4087 (4)	0.74050 (19)	−0.0107 (3)	0.0178 (6)	
H1	0.4160	0.7079	−0.1068	0.021*	
C1	0.4249 (4)	0.6702 (3)	0.1191 (4)	0.0218 (7)	
H1A	0.4410	0.5962	0.1038	0.026*	
C2	0.4176 (5)	0.7076 (2)	0.2761 (4)	0.0206 (7)	
H2A	0.4295	0.6597	0.3677	0.025*	
N2	0.3889 (4)	0.8933 (2)	0.5892 (4)	0.0318 (7)	
C3	0.3922 (4)	0.8184 (2)	0.2937 (4)	0.0164 (7)	
C4	0.3754 (4)	0.8896 (2)	0.1569 (4)	0.0198 (7)	
H4A	0.3575	0.9640	0.1682	0.024*	
C5	0.3857 (4)	0.8479 (2)	0.0032 (4)	0.0193 (7)	
H5A	0.3767	0.8941	−0.0898	0.023*	
C6	0.3875 (5)	0.8612 (3)	0.4580 (4)	0.0202 (7)	

### Atomic displacement parameters (Å^2^)

**Table:**

	*U*^11^	*U*^22^	*U*^33^	*U*^12^	*U*^13^	*U*^23^
Br1	0.0246 (2)	0.01924 (19)	0.01933 (18)	−0.00107 (15)	0.01284 (14)	−0.00215 (13)
N1	0.0149 (14)	0.0239 (14)	0.0174 (12)	0.0034 (11)	0.0094 (11)	−0.0039 (11)
C1	0.024 (2)	0.0189 (17)	0.0243 (17)	0.0035 (14)	0.0111 (15)	−0.0006 (14)
C2	0.0201 (18)	0.0237 (17)	0.0206 (16)	0.0045 (14)	0.0106 (14)	0.0037 (14)
N2	0.045 (2)	0.0311 (17)	0.0251 (15)	−0.0001 (15)	0.0200 (14)	−0.0045 (13)
C3	0.0082 (16)	0.0260 (17)	0.0163 (15)	0.0014 (13)	0.0060 (13)	0.0006 (13)
C4	0.0194 (18)	0.0212 (17)	0.0221 (16)	0.0005 (14)	0.0118 (14)	−0.0032 (13)
C5	0.0166 (18)	0.0226 (17)	0.0207 (16)	0.0007 (14)	0.0094 (14)	0.0034 (14)
C6	0.0184 (18)	0.0248 (17)	0.0194 (16)	−0.0007 (14)	0.0093 (14)	0.0022 (14)

### Geometric parameters (Å, º)

**Table:**

N1—C1	1.337 (4)	N2—C6	1.138 (4)
N1—C5	1.337 (4)	C3—C4	1.387 (4)
N1—H1	0.9003	C3—C6	1.453 (4)
C1—C2	1.381 (4)	C4—C5	1.384 (4)
C1—H1A	0.9300	C4—H4A	0.9300
C2—C3	1.385 (4)	C5—H5A	0.9300
C2—H2A	0.9300		
			
C1—N1—C5	122.9 (2)	C2—C3—C6	120.0 (3)
C1—N1—H1	112.9	C4—C3—C6	119.4 (3)
C5—N1—H1	124.2	C5—C4—C3	118.6 (3)
N1—C1—C2	120.0 (3)	C5—C4—H4A	120.7
N1—C1—H1A	120.0	C3—C4—H4A	120.7
C2—C1—H1A	120.0	N1—C5—C4	119.5 (3)
C1—C2—C3	118.4 (3)	N1—C5—H5A	120.2
C1—C2—H2A	120.8	C4—C5—H5A	120.2
C3—C2—H2A	120.8	N2—C6—C3	178.0 (4)
C2—C3—C4	120.6 (3)		

### Hydrogen-bond geometry (Å, º)

**Table:**

*D*—H···*A*	*D*—H	H···*A*	*D*···*A*	*D*—H···*A*
N1—H1···Br1^i^	0.90	2.26	3.133 (2)	164
C1—H1*A*···Br1	0.93	2.88	3.615 (2)	137
C2—H2*A*···Br1^ii^	0.93	2.77	3.645 (2)	156
C5—H5*A*···N2^iii^	0.93	2.66	3.435 (4)	142

Symmetry codes: (i) −*x*+1, −*y*+1, −*z*; (ii) −*x*+1, −*y*+1, −*z*+1; (iii) *x*, *y*, *z*−1.
